# Genetic Mapping of Resistance in Hexaploid Wheat for a Quarantine Disease: Karnal Bunt

**DOI:** 10.3389/fpls.2018.01497

**Published:** 2018-10-16

**Authors:** Gurcharn S. Brar, Guillermo Fuentes-Dávila, Xinyao He, Carolina P. Sansaloni, Ravi P. Singh, Pawan K. Singh

**Affiliations:** ^1^International Maize and Wheat Improvement Centre (CIMMYT), Texcoco, Mexico; ^2^Department of Plant Science, Crop Development Centre, University of Saskatchewan, Saskatoon, SK, Canada; ^3^Instituto Nacional de Investigaciones Forestales Agricolas y Pecuarias, Mexico City, Mexico

**Keywords:** Karnal bunt, polygenic resistance, QTL, quarantine disease, wheat

## Abstract

Karnal bunt (KB) of wheat, caused by *Tilletia indica*, is one of the greatest challenges to grain industry, not because of yield loss, but quarantine regulations that restrict international movement and trade of affected stocks. Genetic resistance is the best way to manage this disease. Although several different sources of resistance have been identified to date, very few of those have been subjected to genetic analyses. Understanding the genetics of resistance, characterization and mapping of new resistance loci can help in development of improved germplasm. The objective of this study was to identify and characterize resistance loci (QTL) in two independent recombinant inbred lines (RILs) populations utilizing different wheat lines as resistance donors. Elite CIMMYT wheat lines Blouk#1 and Huirivis#1 were used as susceptible female parents and WHEAR/KUKUNA/3/C80.1/3^∗^BATAVIA//2^∗^WBLL1 (WKCBW) and Mutus as moderately resistant male parents in Pop1 and Pop2 populations, respectively. Populations were evaluated for KB resistance in 2015–16 and 2016–17 cropping seasons at two seeding dates (total four environments) in Cd. Obregon, Mexico. Two stable QTL from each population were identified in each environment: *QKb.cim-2B* and *QKb.cim-3D* (Pop1), *QKb.cim-3B1* and *QKb.cim-5B2* (Pop2). Other than those four QTL, other QTL were detected in each population which were specific to environments: *QKb.cim-5B1, QKb.cim-6A*, and *QKb.cim-7A* (Pop1), *QKb.cim-3B2, QKb.cim-4A1, QKb.cim-4A2, QKb.cim-4B, QKb.cim-5A1, QKb.cim-5A2*, and *QKb.cim-7A2* (Pop2). Among the four stable QTL, all but *QKb.cim-3B1* were derived from the resistant parent. *QKb.cim-2B* and *QKb.cim-3D* in Pop1 and *QKb.cim-3B1* and *QKb.cim-5B2* in Pop2 explained 5.0–11.4% and 3.3–7.1% phenotypic variance, respectively. A combination of two stable QTL in each population reduced KB infection by 24–33%, respectively. Transgressive resistant segregants lines derived with resistance alleles from both parents in each population were identified. Single nucleotide polymorphism (SNP) markers flanking these QTL regions may be amenable to marker-assisted selection. The best lines from both populations (in agronomy, end-use quality and KB resistance) carrying resistance alleles at all identified loci, may be used for inter-crossing and selection of improved germplasm in future. Markers flanking these QTL may assist in selection of such lines.

## Introduction

Karnal bunt (KB) of wheat, caused by the fungus *Tilletia indica* Mitra [syn. *Neovossia indica* (Mitra) Mundkur], is one of the greatest challenges to the wheat grain industry. Yield loss is usually limited by this disease, but quality deterioration is a concern because 1–4% of kernel infection is sufficient to make wheat grain unpalatable and 5% of kernel infection causes a distinct deterioration in flour quality (Rush et al., 2005). Additionally, quarantine regulations restrict international movement of KB infected grains from endemic areas (Rush et al., 2005). Other than common wheat (*Triticum aestivum* L.), the disease also affects durum wheat (*T. durum*) and Triticale (x *Triticosecale*). The disease owes its name to Karnal district in India, where it was first reported in 1931, and since has become an important disease in north-western India (Mitra, 1931; Bonde et al., 1997). Apart from India, KB has been reported from Mexico (Duran, 1972), Pakistan (Munjal, 1975), Nepal (Singh et al., 1989), Brazil (Da Luz et al., 1993), the United States of America (APHIS, 1996), Iran (Torarbi et al., 1996), and the Republic of South Africa (Crous et al., 2001). More recently, it has been reported: CIMMYT-blog/tag/karnal-bunt (CIMMYT, 2011), that “Karnal bunt has long been present in Afghanistan, with favorable climatic conditions promoting occasional outbreaks. A recent survey by Agricultural Research Institute of Afghanistan (ARIA) indicated that several popular wheat varieties are susceptible to the disease (Singh, personal communication). It is particularly prevalent in the eastern region bordering Pakistan, which has emerged in recent years as an important seed-producing area within Afghanistan. Despite this, there is no public information regarding the history, disease incidence and the area affected of Karnal bunt in that country. The disease had its worst epiphytotics in North America in 1983, 1985 and 1986 in southern Sonora, Mexico (Lira, 1991).

This floret-infecting smut to bunt fungi damages the developing kernels where it produces black teliospores in sori and can be seed- and/or soil-borne with air-borne sporidial stage (Carris et al., 2006). Fungal teliospores are largely resistant to physical or most chemical treatments, thus making it difficult to manage (Mujeeb-Kazi et al., 2006). Conventional approaches to manage the disease, include cultural practices such as crop rotation, using healthy seed, and adjustment in irrigation timing to avoid infection at susceptible crop growth stages (Singh et al., 2003). Although the disease can be managed by cultural measures and fungicide application to some extent, these approaches fail to provide complete control of the disease which is required to overcome quarantine restrictions (Sansford et al., 2008; Bala et al., 2015). The seed- and soil-borne nature of the causal agent of this disease makes cultural tools ineffective and application of fungicides at anthesis stage might be effective, but not economical for growers (Mujeeb-Kazi et al., 2006; Bala et al., 2015). Karnal bunt can be best managed by exploiting genetic resistance present in wheat and its relatives (Sharma et al., 2004). Resistance to KB has been identified in cultivated as well as wild wheat relatives, although main sources of resistance originate from India, China, Brazil and some lines from the United States (Warham et al., 1986; Fuentes-Dávila and Rajaram, 1994; Villareal et al., 1995, 1996). After screening of several lines, HD29, W485, KB 2012–03 (in PBW343 background), ALDAN “S”/IAS 58, and H 567.71/3^∗^PAR were identified to carry stable resistance in multiple environments in India (Aujla et al., 1992; Sharma et al., 2005; Kumar et al., 2016). Additionally, most accessions of *Aegilops* spp. were found to be resistant to KB (Warham et al., 1986; Chhuneja et al., 2008). Mujeeb-Kazi et al. (2006) also reported resistance in synthetic hexaploid wheats derived from *Aegilops tauschii* and elite durum wheat cultivars and crosses of synthetic hexploids with bread wheat cultivars.

Despite the fact that resistant sources have been identified in the primary wheat gene pool, only a few have been subjected to genetic analyses (Sharma et al., 2005; Mondal et al., 2016). Earlier studies on genetics of resistance have indicated the presence of oligogenic rather than monogenic resistance, which complicates the introgression of multiple genes in elite cultivars (Fuentes-Dávila et al., 1995; Villareal et al., 1995; Sharma et al., 2005). Fuentes-Dávila et al. (1995) identified six resistance genes *Kb1, Kb2, Kb3, Kb4, Kb5*, and *Kb6* in six resistant sources originating from China (Shanghai), Brazil (PF71131), the United States (Chris), and Mexico (Amsel, CMH77.308, Pigeon). They further reported the effectiveness of combining dominant or partially dominant genes to reduce KB infection levels in wheat. Singh et al. (2007) identified three QTL from HD29 and W485 using molecular mapping approach, which explained 13–19% phenotypic variation in the mapping populations. Additionally, Sharma et al. (2005) identified two resistance genes in each of HD29, W485, and ALDAN “S”/IAS 58 and three in H 567.71/3^∗^PAR in their genetic study.

Although, identification and mapping of major resistance loci from highly resistant wheat lines are important, the lines carrying multiple minor QTL or moderately resistant genotypes for genetic characterization should not be ignored. Advanced breeding lines with favorable agronomic, end-use quality and disease resistant traits should be ideal candidates for gene mapping as such material can easily be used and exploited in wheat breeding programs. Also approaches to screen for KB resistance are time-consuming and labor intensive as the inoculations are performed at the boot stage and disease rating is performed after harvest by counting infected kernels. Disease escape is not uncommon either. In such scenarios, development of breeder friendly markers for KB resistance loci could be useful in efficient introgression of QTL in breeding material. Breeding for KB resistance is not only important for countries where the pathogen exist, but for as many as 70 countries that are imposing quarantine regulations on movement of grain from affected countries (Chhuneja et al., 2008). The present study was aimed at characterizing resistance loci in two recombinant inbred lines (RILs) populations derived from elite breeding lines from CIMMYT’s wheat breeding program.

## Materials and Methods

### Plant Materials

Two bi-parental mapping populations utilizing different elite lines as resistance donors were used in the study. The first bi-parental population (named Pop1) was derived from a cross between CIMMYT breeding lines Blouk#1 and WHEAR/KUKUNA/3/C80.1/3^∗^BATAVIA//2^∗^WBLL1 (WKCBW hereafter). The RILs were developed through single seed descent and advanced to the F_6_ generation, with 165 progeny. The female parent Blouk #1 is a KB susceptible line, whereas the male parent WKCBW is a moderately resistant line, showing consistently better resistance than the female parent in several previous experiments. The second mapping population (named Pop2) comprised of 275 F_6_ RILs and was developed using similar approach as Pop1. In Pop2, Huirivis#1 was used as a female parent (moderately susceptible) and Mutus as the male parent and resistance donor (resistant to moderately resistant).

### Karnal Bunt Inoculations and Disease Ratings

Field experiments for KB disease screening were conducted at the Norman E. Borlaug Experimental station (CENEB) station in Cd. Obregon, Mexico, during the 2015–16 and 2016–17 cropping seasons. The populations were seeded on two dates, with the first being mid-November and the second 2 weeks later, thus resulting in four environments. Recombinant inbred lines were seeded in 1 m double row plots spaced 20 cm apart, on 75 cm wide raised beds. The nursery was placed under protective mesh to prevent bird damage. The KB susceptible check WL711 was seeded in a range of dates to monitor disease pressure over the whole experiment period. Field management followed recommended local practices.

For inoculum preparation, KB infected wheat kernels were mixed with Tween 20 solution in a glass tube and shaken, then the solution was filtered with 60 μm mesh and allowed to stand for 24 h. The collected teliospores were placed in 0.6% sodium hypochlorite for 2 min and centrifuged shortly at 3,000 rpm. The supernatant was discarded and distilled water was added to rinse the teliospores, then the solution was briefly centrifuged at 3,000 rpm and the rinse and centrifuge steps were repeated once more. The teliospores were transferred to 2% water agar under sterile condition and incubated at 18–22°C until germination was detected. Pieces of water agar with teliospores germinating on it were put inversely onto Petri dishes with potato-dextrose-agar (PDA) in order to stimulate the production of secondary sporidia. Nine days later, the Petri dishes were flooded with sterile water, scraped with a sterile spatula, and the suspension was transferred to other Petri dishes with PDA to increase the inoculum. Once the Petri dishes were covered with fungal colonies, the agar was cut into pieces and put inversely on sterile glass Petri dishes, into which distilled water was added and secondary sporidia were daily collected. Inoculum concentration was adjusted to 10,000 sporidia/ml using a haemocytometer. Inoculations were done via injecting 1 ml of inoculum into the boot at the booting stage, and five randomly selected spikes were inoculated in each line. To maintain high humidity in the screening nursery, an automated misting system was equipped and the sprinklers sprayed five times per day for 20 min each time. The inoculated spikes were separately harvested and manually threshed, then infected and total grain numbers were scored for each spike for the calculation of disease severity. Averaged KB severity over the five spikes were used in subsequent analyses.

### Genotyping, QTL Mapping

Genomic DNA was extracted from young leaves following the previously described CTAB method (Osman et al., 2015). The populations were genotyped with the DArTseq technology at the Genetic Analysis Service for Agriculture (SAGA) at CIMMYT, Mexico. This genotyping platform is based on a combination of complexity reduction methods developed for array-based DArT and sequencing of resulting representations on next-generation sequencing platforms, as described in detail in Li et al. (2015).

Single nucleotide polymorphisms (SNPs) loci with both alleles were used in the genetic mapping. The SNP loci with missing values in > 20% and deviating from expected 1:1 ratio (based on Chi-square test) were discarded. Genetic linkage maps were constructed using JoinMap ver. 4.0 software (Van Ooijen and Voorrips, 2004) with a minimum logarithm of the odds ration (LOD) score of 3.0 using “Haldane” mapping function. The maximum likelihood approach was used to order markers in each linkage group. Linkage groups were assigned chromosome names based on published DArT markers’ consensus map (Li et al., 2015). Least square means of the phenotypic data were used in the QTL mapping and analyses. The QTL analyses were performed using percent KB infection in four environments, i.e., two cropping seasons and the two seeding dates as well as a combined QTL analyses on averaged phenotypic data across all four environments resulting into a total of five environments. A simple interval mapping (IM) model was used to identify markers associated with the trait followed by the multiple QTL mapping (MQM) approach using the closest linked markers to each QTL (detected using IM) as co-factors. The minimum LOD score was set to 2.5 for QTL mapping, but in some environments the permutation tests indicated LOD > 1.6 as the level of significance of linkage (Van Ooijen, 1999).

### Physical Mapping and Annotation

All SNP markers flanking or in the QTL intervals were physically positioned on the Chinese Spring wheat genome sequence available through International Wheat Genome Sequencing Consortium. The SNP-bearing sequences were probed to the entire bread wheat NRGene genome assembly ver. 1.0 (International Wheat Genome Sequencing Consortium)^1^ using an in-house BLAST portal. The best hits, based on sequence similarity and cumulative alignment length percentage of matches, were considered. For annotation, the flanking marker sequences were used to find expressed genes on the scaffolds using POTAGE (PopSeq Ordered *Triticum aestivum* Gene Expression) (Suchecki et al., 2017). POTAGE integrates map location with gene expression and inferred functional annotation and visualizes these data through a web browser interface.

### Statistical Analyses and QTL Haplotype Analyses

Phenotypic data from four environments was subjected to analysis of variance (ANOVA) to partition variation among manipulated factors. Except, Genotype/RIL entry/QTL haplotype group, all other factors were considered random. Assumptions of normality and homogeneity of error variance for all class variables was estimated using Shapiro–Wilk’s and Levene’s tests, respectively, implemented in the procedure UNIVARIATE in the Statistical Analysis Software (SAS) ver. 9.4. Heterogeneous variances in class variables, if any, were modeled using repeated/group = effect statement in procedure MIXED (Littell et al., 2006). The least significant difference (LSD) was calculated according to Fisher’s method with DDFM = kenwardroger option for approximating the degrees of freedom for means. All tests used a nominal alpha level of 0.05. Broad sense-heritability (*h*^2^) was calculated using variance components of different variables as follows: $H^{2}={\text{σ}}_{g}^{2}/\left({\text{σ}}_{g}^{2}+\frac{{\text{σ}}_{g*E}^{2}}{E}+\frac{{\text{σ}}_{e}^{2}}{E.r}\right),$ where ${\text{σ}}_{g}^{2}$ is genotypic variance due to RILs, ${\text{σ}}_{g*E}^{2}$ is genotype by environment interaction variance, ${\text{σ}}_{g}^{2}$ is error variance, *E* is number of environments, *r* is number of replications. Variance components were estimated using procedure VARCOMP in SAS ver. 9.4 with the restricted maximum likelihood method. Correlation coefficients among all traits were also calculated using the procedure CORR in SAS ver. 9.4. Associations among environments, genotypes, and genotype by environment interaction were also analyzed and visualized using biplot analyses (Yan and Tinker, 2006) in the R environment using GGEBiplotGUI package (Frutos et al., 2014). For biplot analyses, following settings were used: singular value portioning- environment-metric preserving; genotype by environment scaling- according to standard deviation; centered by environment (G + G^∗^E). The effect of combining alternate alleles at identified QTL loci was tested on both populations. However, only stable QTL were used for haplotype analyses. For QTL–QTL interaction/haplotype analyses, the closest markers to each stable QTL were used to assign a QTL class to entries. Least squares’ means were calculated for each QTL class using the procedure MIXED in SAS ver. 9.4 and the means comparison was performed as was done using Fisher’s LSD (least significant difference) at *P* = 0.05. In context of this paper, stable QTL are the ones which were detected in all environments for Pop1 and in at least four of the five environments in Pop2.

## Results

### Phenotypic Evaluation and Transgressive Segregation of RILs

**FIGURE 1:**
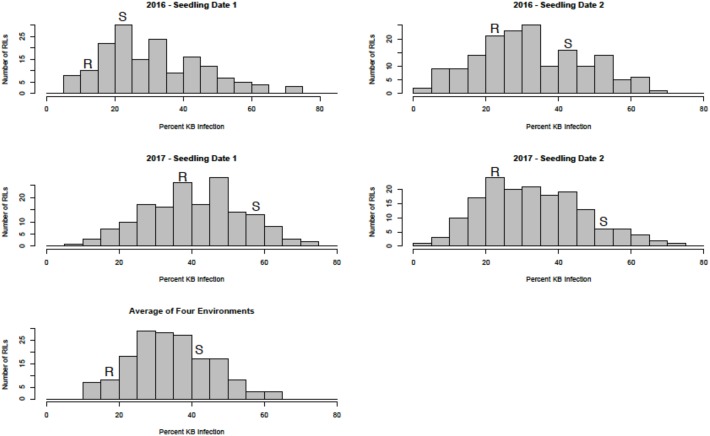
Distribution of Karnal bunt infection over different environments in recombinant inbred lines (RILs) from Pop1 (Blouk#1/WKCBW). Blouk#1 was used as a female and susceptible (S) parent and WKCBW as resistant (R) parent.

**FIGURE 2:**
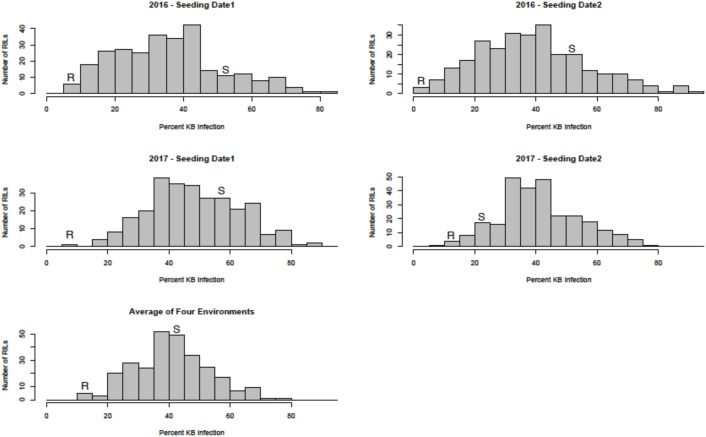
Distribution of Karnal bunt infection over different environments in recombinant inbred lines (RILs) from Pop2 (Huirivis#1/Mutus). Huirivis#1 was used as a female and susceptible (S) parent and Mutus as resistant (R) parent.

**FIGURE 3:**
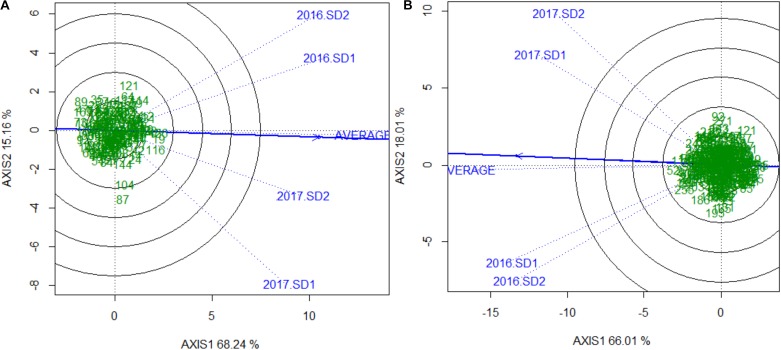
Genotype and genotype by environment (GGE) interaction plot showing relationship among genotypes, environments and their interaction for **(A)** Pop1 (Blouk#1/WKCBW) and **(B)** Pop2 (Huirivis#1/Mutus). Numbers in the green indicates NIL entries and blue labels and vectors are unique to every environment. The solid blue line passing through the origin of the plot is “Average Environment Axis” indicating the most ideal and discriminating environment. The axes of the plot indicate standard deviation for phenotype (proportional to length of environment vector). The phenotypic variation explained by both axis is indicated next to the labels.

Sufficient disease pressure was observed in all environments and was evidenced by the significant positive correlation of percent KB infection among environments (**Table 1**). For both populations, the resistant parents showed lower seed infection when compared to the susceptible parent (**Figure 1**). For both populations, the maximum range of infection remained above 60% (**Figures 1**, **2** and **Table 3**). The distribution for percent KB infection was continuous and skewed for higher infection in most of the environments in both populations. Effects of genotypes/RILs and their interaction with the environment were significant in both populations whereas environment did not significantly influence the percent KB infection (**Table 2**). Association among genotypes and environments was also studied using biplot analyses and the environments showed positive correlation as indicated by the acute angle among environment vectors (**Figure 3**). Although there was significant correlation among all environments, as indicated by biplot analyses and correlation coefficients, correlation among seeding dates in any given environment indicated a higher seasonal variability compared to seeding date (**Table 1** and **Figure 3**). Longer vectors for all environments indicated sufficient disease pressure to distinguish RILs and all RIL entries were grouped near the origin of the plots indicating non-significant environmental influence in corroboration with ANOVA results (**Table 2** and **Figure 3**). Notably, broad-sense heritability was high (> 0.70) in both populations (**Table 2**). Transgressive segregation was observed in both populations, although a relatively small proportion of RILs showed better resistance than the parents (**Figures 1**, **2**). A small proportion of RILs in both populations showed < 15% seed infection in all environments. For averaged infection over environments, both parents for Pop1 and Pop2 had lower infection as compared to the susceptible check line WL711 (**Table 3**). However, none of the parents were comparable to resistant check Munal#1, which was almost immune to KB infection. Although resistant transgressive segregants were better than their respective resistant parents, none of the RILs was comparable to Munal#1.

**Table 1:** Pearson correlation coefficients among environments for percent Karnal bunt infection in Pop1 (BLOUK#1/WKCBW) and Pop2 (Huirivis#1/Mutus).

	2016-SD1	2016-SD2	2017-SD1	2017-SD2	Average
**POP1**					
2016-SD1	–				
2016-SD2	0.71^∗^	–			
2017-SD1	0.46^∗^	0.30^∗^	–		
2017-SD2	0.41^∗^	0.48^∗^	0.44^∗^	–	
Average	0.84^∗^	0.81^∗^	0.70^∗^	0.75^∗^	–
**POP2**					
2016-SD1	–				
2016-SD2	0.71^∗^	–			
2017-SD1	0.46^∗^	0.38^∗^	–		
2017-SD2	0.35^∗^	0.35^∗^	0.57^∗^	–	
Average	0.83^∗^	0.82^∗^	0.75^∗^	0.69^∗^	–

*^∗^Represent statistical significance of correlation coefficient at P = 0.05.*

**Table 2:** Analysis of variance for Karnal bunt infection (%) of the recombinant inbred lines (RILs) in Pop1 (BLOUK#1/WKCBW) and Pop2 (Huirivis#1/Mutus) and broad-sense heritability (*H*^2^) estimates.

Source of variation	Degree of freedom (df)	*F-*value	*P-*value	*H*^2^
Pop1				0.78
Genotype (RILs)	164	7.56	0.0001	.
Environment (E)	4	1.37	0.0856	.
Genotype^∗^E	656	17.89	0.0001	.
Error		.^a^	.	.
Pop2				0.75
Genotype (RILs)	274	7.50	0.0001	.
Environment (E)	4	1.39	0.0817	.
Genotype^∗^E	1096	23.15	0.0001	.
Error		.	.	.

*^*a*^Variation due to error term was small and thus was not estimated by the SAS program.*

**Table 3:** Descriptive statistics on percent Karnal bunt (KB) infection in parents of Pop1 and Pop2, check lines, and recombinant inbred lines (RILs).

	Pop1 (Blouk#1/WKCBW)^a^	Pop2 (Huirivis#1/Mutus)^b^
Minimum (among RILs)	11.8	12.8
Maximum (among RILs)	62.7	79.2
Susceptible parent	39.5 (Blouk#1)	42.2 (Huirivis#1)
Resistant parent	17.4 (WKCBW)	4.6 (Mutus)
WL711 (susceptible check)	46.3	46.3
Munal#1 (resistant check)	2.3	2.3

*The percent KB infection is averaged over all tested environments.^*a*^Blouk#1 and WKCBW are susceptible and moderately resistant parents, respectively. ^*b*^Huirivis#1 and Mutus are moderately susceptible and resistant parents, respectively.*

### Marker Analyses

After filtering for missing data and segregation distortion, a total of 558 and 500 skeleton SNP markers were used for linkage mapping in Pop1 and Pop2, respectively. There was one marker in Pop1 and two in Pop2, which could not be assigned to any linkage group. Pop1 and Pop2 were divided into a total of 27 and 28 linkage groups, respectively. Marker order in linkage groups was generally in agreement with published consensus map (Li et al., 2015). In Pop1, no marker could be assigned to chromosome 5D. Total map lengths for Pop1 and Pop2 was 1432.8 and 1656.7 cM, respectively. In Pop1, genomes A, B, and D were assigned 285 (51.2%), 213 (38.2%), and 59 (10.6%) markers, whereas in Pop2 this was 305 (61.2%), 154 (30.9%), and 39 (7.8%) markers. The majority of markers in both populations were assigned to the A and B genomes.

### QTL Mapping and Haplotype Analyses

#### Pop1

**FIGURE 4:**
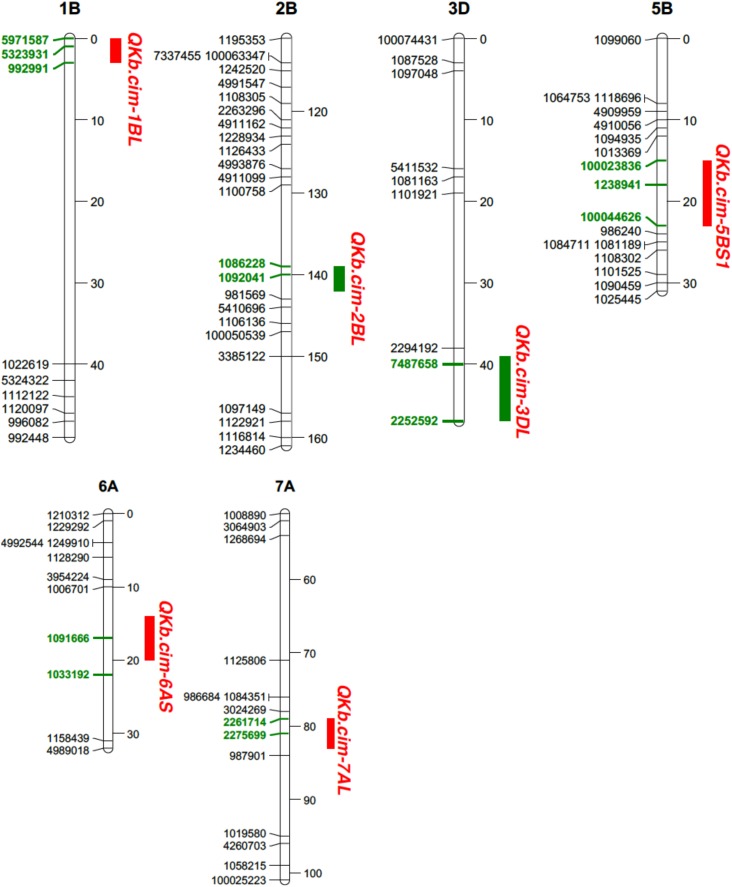
Portion of genetic linkage maps of QTL conferring resistance to Karnal bunt infection in Pop1 (Blouk#1/WKCBW). Blouk#1 was used as a female and susceptible parent and WKCBW as resistant parent. Values to the right of the chromosomes indicate genetic distance (cM) of map. QTL intervals depicted in green bars were detected in all environments. Markers in boldface and green are associated with the QTL.

In Pop1, six different QTL were mapped on chromosomes 1B, 2B, 3D, 5B, 6A, and 7A: *QKb.cim-1BL, QKb.cim-2BL, QKb.cim-3DL, QKb.cim-5BS1, QKb.cim-6AS*, and *QKb.cim-7AL* (**Figure 4**). The flanking SNP markers (Clone ID) for all identified QTL are presented in **Table 3**. The LOD values in the QTL regions varied among environments, but all the QTL reported in this study demonstrate a significant linkage with percent KB infection. Except *QKb.cim-5BS1* and *QKb.cim-6AS*, all others were derived from resistant parent WKCBW (**Table 4**). Only *QKb.cim-2BL* and *QKb.cim-3DL* were identified in all four environments and others were identified only in one to three environments. QTL in Pop1 explained 5.0–11.4% of phenotypic variance (**Table 4**), and were mapped within 5 cM interval, except *QKb.cim-3DL* which was flanked by markers about 6 cM apart (**Figure 4**). **Table 5** shows the effect of combining alternate alleles at *QKb.cim-2BL* and *QKb.cim-3DL* haplotypes in Pop1. It is evident that both QTL are equally effective when introgressed singly and significantly better than susceptible alleles at both loci in terms of disease reduction. A combination of both QTL is better than either single QTL or susceptible alleles at both loci. The combination of both QTL is capable of reducing percent KB infection by up to 33% (**Table 6**).

**Table 4:** Quantitative trait loci (QTL) associated with Karnal bunt infection in Pop1 (Blouk#1/WKCBW) in different environments.

Environment^a^	QTL	Flanking markers	Chromosome	Source of resistance allele	LOD^b^ score	PVE^c^
2016-SD1	*QKb.cim-2BL*	1092041–1086228	2B	WKCBW	2.7	7.0
	*QKb.cim-3DL*	7487658–2252592	3D	WKCBW	2.5	6.7
2016-SD2	*QKb.cim-3DL*	7487658–2252592	3D	WKCBW	3.4	9.0
2017-SD1	*QKb.cim-2BL*	1092041–1086228	2B	WKCBW	3.5	9.2
	*QKb.cim-3DL*	2294192–2252592	3D	WKCBW	1.8	5.0
	*QKb.cim-7AL*	2261714–2275699	7A	WKCBW	2.3	6.3
	*QKb.cim-1BL*	5323931–992991	1B	WKCBW	2.8	7.5
2017-SD2	*QKb.cim-2BL*	1092041–1086228	2B	WKCBW	3.1	8.3
	*QKb.cim-3DL*	7487658–2252592	3D	WKCBW	1.9	5.2
	*QKb.cim-5BS1*	100044626–100023836	5B	Blouk#1	2.1	5.6
	*QKb.cim-6AS*	1033192–1091666	6A	Blouk#1	2.0	5.4
Average	*QKb.cim-2BL*	1092041–1086228	2B	WKCBW	4.3	11.4
	*QKb.cim-3DL*	2294192–2252592	3D	WKCBW	4.0	10.5
	*QKb.cim-5BS1*	100044626–100023836	5B	Blouk#1	2.2	6.0

*^*a*^Here: SD1, seeding date 1; SD2, seeding date 2; Average, average of four environments.^*b*^Logarithm of odds score from the peak of the QTL.^*c*^Phenotypic variation explained (%).*

**Table 5:** Quantitative trait loci (QTL) associated with Karnal bunt infection in Pop2 (Huirivis#1/Mutus) in different environments.

Environment^a^	QTL	Flanking markers	Chromosome	Source of resistance allele	LOD^b^ score	PVE^c^
2016-SD1	*QKb.cim-3BS1*	100010977–1079551	3B	Huirivis#1	2.5	4.1
	*QKb.cim-3BS2*	5969907–4989073	3B	Huirivis#1	2.3	3.8
	*QKb.cim-5BS2*	2253589–1011847	5B	Mutus	2.1	3.5
	*QKb.cim-7AS*	100013417–1218489	7A	Huirivis#1	2.5	4.1
2016-SD2	*QKb.cim-3BS1*	100010977–1079551	3B	Huirivis#1	2.0	3.3
	*QKb.cim-3BS2*	5969907–4989073	3B	Huirivis#1	2.1	3.5
	*QKb.cim-4AS1*	100010444–1204980	4A	Mutus	3.2	5.2
	*QKb.cim-4AS2*	3023036–1213856	4A	Huirivis#1	4.5	7.2
	*QKb.cim-5BS2*	2253589–1011847	5B	Mutus	2.2	3.7
2017-SD1	*QKb.cim-3BS1*	100010977–1079551	3B	Huirivis#1	3.4	5.5
	*QKb.cim-4AS1*	7337280–1204980	4A	Mutus	2.0	3.3
	*QKb.cim-5AL1*	2253865–1013608	5A	Mutus	2.5	4.0
	*QKb.cim-5AL2*	5411517–1146968	5A	Mutus	2.6	4.3
	*QKb.cim-5BS2*	2253589–1011847	5B	Mutus	4.4	7.1
2017-SD2	*QKb.cim-3BS1*	100010977–1079551	3B	Huirivis#1	2.6	4.3
	*QKb.cim-4BL*	1132777–1863994	4B	Mutus	2.7	4.5
	*QKb.cim-5BS2*	2253589–1011847	5B	Mutus	2.4	4.0
Average	*QKb.cim-3BS1*	100010977–1079551	3B	Huirivis#1	4.2	6.7
	*QKb.cim-3BS2*	5969907–4989073	3B	Huirivis#1	2.8	4.5
	*QKb.cim-4AS1*	100010444–1204980	4A	Mutus	2.9	4.7
	*QKb.cim-4AS2*	3023036–1213856	4A	Huirivis#1	2.8	4.6
	*QKb.cim-5BS2*	2253589–1011847	5B	Mutus	4.2	6.7

*^*a*^Here: SD1, seeding date 1; SD2, seeding date 2; Average, average of four environments.^*b*^Logarithm of odds score from the peak of the QTL.^*c*^Phenotypic variation explained (%).*

**Table 6:** Effects of combining alternate alleles at stable QTL on percent Karnal bunt (KB) infection (combined over all environments) in Pop1 and Pop2.

QTL for KB resistance^a^		Mean^b^	Reduction (%)
**POP1 (BLOUK#1/WKCBW)^c^**			
***QKb.cim-2BL***	***Q.Kb.cim-3DL***		
Blouk#1	Blouk#1	41.8a (*n* = 37)^e^	–
Blouk#1	WKCBW	34.4b (*n* = 18)	17.7
WKCBW	Blouk#1	32.9b (*n* = 24)	21.3
WKCBW	WKCBW	27.8c (*n* = 33)	33.5
**POP2 (HUIRIVIS#1/MUTUS)^d^**			
***QKb.cim-3BS1***	***QKb.cim-5BS2***		
Huirivis#1	Huirivis#1	42.3b (*n* = 54)	7.0
Huirivis#1	Mutus	34.2c (*n* = 49)	24.8
Mutus	Huirivis#1	45.5a (*n* = 66)	–
Mutus	Mutus	40.4b (*n* = 46)	11.2

*^*a*^QTL QKb.cim-2BL and QKb.cim-3DL are derived from KCBW. QTL QKb.cim-3BS1 and QKb.cim-5BS2 are derived from Huirivis#1 and Mutus, respectively.^*b*^Means followed by the same letter are not statistically significantly different according to Fisher’s least significant difference (LSD) at P = 0.05. ^*c*^Blouk#1 and WKCBW are susceptible and moderately resistant parents, respectively.^*d*^Huirivis#1 and Mutus are moderately susceptible and resistant parents, respectively.^*e*^Number of recombinant inbred lines carrying that haplotype*.

#### Pop2

**FIGURE 5:**
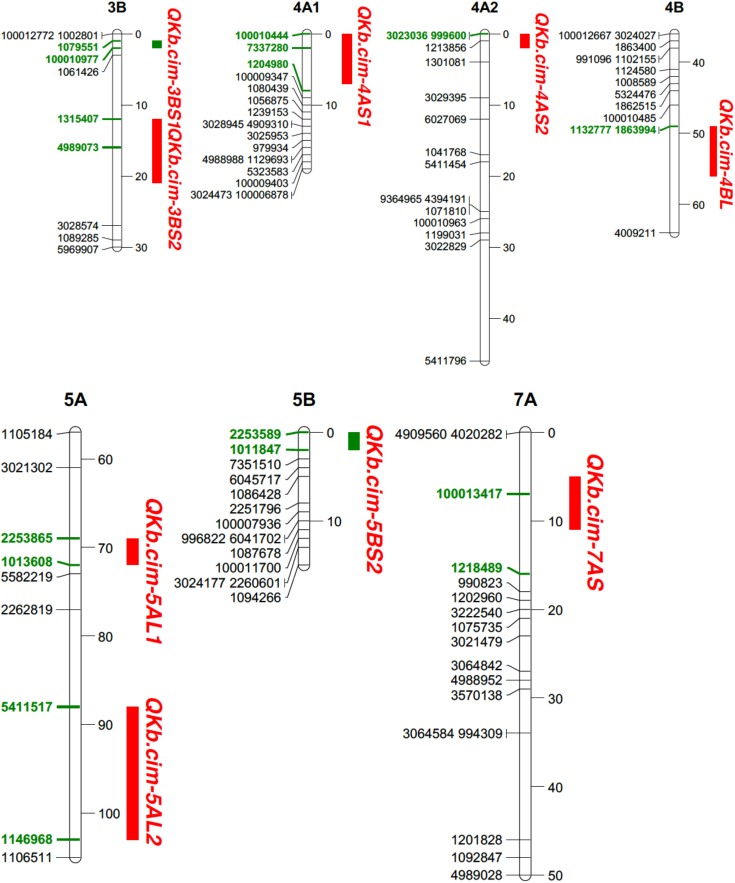
Portion of genetic linkage maps of QTL conferring resistance to Karnal bunt infection in Pop2 (Huirivis#1/Mutus). Huirivis#1 was used as a female and susceptible parent and Mutus as resistant parent. Values to the right of the chromosomes indicate genetic distance (cM) of map. QTL intervals depicted in green bars were detected in all environments. Markers in boldface and green are associated with the QTL.

Relatively more QTL were detected in Pop2 (**Table 5**). In Pop2, nine QTL on chromosomes 3B, 4A, 4B, 5A, 5B, and 7A were detected: *QKb.cim-3BS1, QKb.cim-3BS2, QKb.cim-4AS1, QKb.cim-4AS2, QKb.cim-4BL, QKb.cim-5AL1, QKb.cim-5AL2, QKb.cim-5BS2*, and *QKb.cim-7AS* (**Figure 5** and **Table 5**). The flanking SNP markers for all identified QTL are reported in **Table 4**. QTL *QKb.cim-3BS1, QKb.cim-3BS2, QKb.cim-4AS2*, and *QKb.cim-7AS* were derived from susceptible parent Huirivis#1 and explained 3.3–6.7% of phenotypic variance (**Table 5**). All other QTL were derived from the moderately resistant parent Mutus and explained 3.3–7.1% phenotypic variance. Only two QTL: *QKb.cim-3BS1* and *QKb.cim-5BS2* were identified in at least four of the five environments and were flanked by markers spanning < 5 cM interval (**Figure 5**). The results of haplotype combinations for the two stable QTL were similar to Pop1. The combination of resistance alleles at both stable QTL reduced the percent KB infection by 25% (**Table 6**). It is important to note that the improvement in disease reduction in Pop2 is relatively lower as compared to Pop1.

### Physical Mapping and Predicted Genes in QTL Regions

Physical mapping of SNP markers flanking QTL in both populations confirmed their chromosome and arm assignments (**Table 7**). Except *QKb.cim-3DL* in Pop1, all other QTL spanned less than 12 Mb. The *QKb.cim-3DL* also spanned a large interval on genetic map as compared to other QTL (**Figure 4**). For Pop2, the largest physical interval of 77 Mb was associated with *QKb.cim-4AS1* (**Table 7**). All the marker sequences that flanked the QTL intervals were used for BLASTn searches to find expressed genes in the region. Number of annotated genes in identified QTL regions ranged between 13 and 478 (**Supplementary Table S1**). Except *QKb.cim-4AS1* and *QKb.cim-4BL*, all other QTL regions had less than 85 expressed genes. Based on the expression level in spike or grain and the potential function of the gene, potential candidate genes for the identified QTL are listed in **Table 8**. A wide variety of genes were expressed in the region but many can easily be eliminated based on their zero expression values in many plant parts, particularly spike and grain (**Supplementary Table S1**).

**Table 7:** Physical position of loci carrying single nucleotide polymorphism (SNP) markers that flank the quantitative trait loci (QTL) in Pop1 and Pop2.

QTL	Flanking markers	Physical position (Mb)^a^
**POP1 (BLOUK#1/WKCBW)**		
*QKb.cim-1BL*	5971587	645.94
	5323931	646.90
	992991	647.20
*QKb.cim-2BL*	1086228	749.99
	1092041	755.59
*QKb.cim-3DL*	7487658	282.96
	2252592	66.25
*QKb.cim-5BS1*	100023836	26.02
	1238941	30.87
	100044626	38.01
*QKb.cim-6AS*	1091666	13.82
	1033192	19.99
*QKb.cim-7AL*	2261714	540.87
	2275699	552.68
**POP2 (HUIRIVIS#1/MUTUS)**		
*QKb.cim-3BS1*	1079551	3.80
	100010977	–^b^
*QKb.cim-3BS2*	1315407	10.66
	4989073	16.70
*QKb.cim-4AS1*	100010444	47.65
	7337280	–
	1204980	125.34
*QKb.cim-4AS2*	3023036	4.24
	1213856	4.59
*QKb.cim-4BL*	1132777	535.10
	1863994	548.19
*QKb.cim-5AL1*	2253865	487.76
	1013608	478.85
*QKb.cim-5AL2*	5411517	545.57
	1146968	553.34
*QKb.cim-5BS2*	2253589	53.80
	1011847	47.59
*QKb.cim-7AS*	100013417	19.91
	1218489	32.37

*^*a*^Physical position was mapped by aligining the sequence against Chinese Spring assembly from International Wheat Genome Sequencing Consortium (IWGSC) RefSeq ver. 1.^*b*^No hit.*

**Table 8:** List of annotated genes for each QTL, with the respective Munich Information Center for Protein Sequences (MIPS) hits and rice annotation hits.

QTL	Gene ID	Gene name/description	MIPS annotation hit	Rice annotation hit
**POP1 (BLOUK#1/WKCBW)**				
*QKb.cim-1BL*	Traes_1BL_408EABE70	Auxin response factor 15	sp| Q8S985| ARFO_ORYSJ	LOC_Os05g48870.1
	Traes_1BL_1091D4522	Protein DEHYDRATION-INDUCED	sp| Q688X9| DI191_ORYSJ	LOC_Os05g48800.1
	Traes_1BL_EE4652640	xyloglucan endotransglucosylase/hydrolase 25	AT5G57550.1 AT5G57550.1	LOC_Os08g13920.1
*QKb.cim-2BL*	Traes_2BL_7D4DBA293	Acyl-transferase family protein	AT3G29670.1	LOC_Os04g54560.1
	Traes_2BL_795807EE4	Protein kinase	AT3G25490.1	LOC_Os10g09620.1
	Traes_2BL_CCD296233	Stress enhanced protein	sp| Q9SJ02| STEP2_ARATH	LOC_Os04g54630.1
	Traes_2BL_05498B97F	ABC transporter	sp| Q9LJX0| AB19B_ARATH	LOC_Os04g38570.1
	Traes_2BL_B04506AD11	Wound-responsive family protein	AT4G10270.1	LOC_Os04g54300.1
*QKb.cim-3DL*	–^a^	–	–	–
*QKb.cim-5BS1*	Traes_5BS_B5A6A3EF7	Disease resistance protein	sp| Q9T048| DRL27_ARATH	LOC_Os12g37770.1
	Traes_5BS_B7F04C1A5	ABC transporter	sp| Q8VZZ4| AB6C_ARATH	LOC_Os01g07870.1
	Traes_5BS_007B4073F	Protein kinase family protein	AT5G02070.1	LOC_Os12g42070.1
	Traes_5BS_3F6023CD6	Auxin response factor 6	AT1G30330.2	LOC_Os12g41950.1
*QKb.cim-6AS*	Traes_6AS_6C330B811	Disease resistance protein RPM1	sp| Q39214| RPM1_ARATH	LOC_Os08g42700.1
	Traes_6AS_CBC883522	Wall-associated receptor kinase 2	sp| Q9LMP1| WAK2_ARATH	LOC_Os02g02120.1
*QKb.cim-7AL*	–	–	–	–
**POP2 (HUIRIVIS#1/MUTUS)**				
*QKb.cim-3BS1*	Traes_3AS_9E9A03E98	Wall-associated protein kinase	sp| Q9LMP1| WAK2_ARATH	LOC_Os04g30010.1
	Traes_3B_E735811B8	Disease resistance protein	sp| Q9T048| DRL27_ARATH	LOC_Os04g35210.1
*QKb.cim-3BS2*	Traes_3B_601B500B5	Glutathione S-transferase family protein	AT1G10370.1	LOC_Os10g38600.1
*QKb.cim-4AS1*	Traes_4AS_35FB8DB6E	Disease resistance protein	sp| Q9T048| DRL27_ARATH	LOC_Os03g14900.1
	Traes_4AS_05D1CEFD5	Pathogenesis-related thaumatin superfamily protein	AT4G38660.1	LOC_Os03g14030.1
	Traes_4AS_D7D3552A7	Glutathione S-transferase family protein	AT5G02790.1	LOC_Os03g17470.1
*QKb.cim-4AS2*	Traes_4AS_F54BA27F7	Protein kinase superfamily protein	AT3G20530.1	LOC_Os03g08550.2
*QKb.cim-4BL*	Traes_4BL_E01EDE97C	4-coumarate:CoA ligase 3	AT1G65060.1	LOC_Os03g05780.1
*QKb.cim-5AL1*	–	–	–	–
*QKb.cim-5AL2*	–	–	–	–
*QKb.cim-5BS2*	Traes_5BS_E7ADA47A4	Pectinesterase family protein	AT3G14300.1	LOC_Os01g21034.1
	Traes_5BS_9F043AE17	NAC domain protein	UniRef90_B9HH13	LOC_Os12g41680.1
	Traes_5BS_0736D4AEA	Glutathione S-transferase Z1	sp| Q9ZVQ3| GSTZ1_ARATH	LOC_Os02g35590.2
*QKb.cim-7AS*	Traes_7AS_643102EBE	Disease resistance protein CC-NBS-LRR class family	AT5G48620.1	LOC_Os06g17880.1

*^*a*^No annotation obtained.*

## Discussion

Karnal bunt is one of those few quarantine plant diseases that hampers free trade among countries due to imposed quarantine regulations (Rush et al., 2005). Thus, it is imperative to breed for KB resistance in wheat so as to allow import-export of one of the most important crop commodities globally. Breeding for a particular trait of interest may not be as simple because wheat breeders tend to pyramid tens of different traits in a desirable background. Thus, even in the existence of a highly resistant germplasm, breeders may not always utilize it due to undesirable alleles (resulting from linkage drag), that can be associated with the resistance loci. Thus, to avoid any potential linkage drag, wheat pathologists and breeders focus on identification/mapping of resistance loci from elite or advanced breeding lines with favorable plant type, agronomic traits and end-use quality traits. Our study used elite breeding lines with differential resistance response to KB from CIMMYT’s wheat breeding program. This was used to identify and map resistance QTL for KB seed infection in two independent populations, utilizing different lines as resistance donors.

The quantitative inheritance of KB resistance in wheat is well-established from previous studies (Singh et al., 2003, 2007; Sharma et al., 2005), and our study corroborate these findings. Karnal bunt disease expression is greatly influenced by prevailing environmental conditions and very specific conditions are required at a particular growth stage for successful infection (Rush et al., 2005). Unlike other wheat bunts, in KB, dikaryotization of compatible mating types in host is not uncommon, and thus, failure of fusion might reduce disease incidence or escapes may be observed even during artificial inoculations (Singh et al., 1999). Although the disease is highly influenced by environmental conditions, in our study the environmental effect was not significant for either population, and that could be why high heritability of the trait was observed. Also, the protocol for KB resistance evaluation is well-established which leads to less environmental or unexplained variation in the KB nursery at CENEB, Cd. Obregon (Fuentes-Dávila et al., 1995). High heritability in our study indicated that the genetic variation in KB resistance is highly heritable. Therefore, the QTL identified in our study have a strong genetic base and are highly heritable.

Of the four stable QTL identified in our study, QTL *QKb.cim-2BL, QKb.cim-3BS1*, and *QKb.cim-5BS2* are flanked by SNP markers less than 5 cM apart and 6 Mb apart according to their physical location. Although we utilized low number of high-quality SNP markers, the promising QTL were mapped within 5 cM. Saturation of the QTL intervals with additional number of SNP markers can help fine-map the QTL and thus making it amenable for breeder-friendly Kompetitive allele-specific PCR (KASP) assays. The use of flanking markers for these QTL may enable marker-assisted selection or introgression into other elite backgrounds. The QTL *QKb.cim-3DL* is approximately 6.5 cM in length of chromosome arm, and fine mapping of the QTL may help narrow down the interval. The additive nature of KB resistance loci is reported in previously published literature, and is evidenced in our study as the combination of QTL was better than any QTL present singly in RILs. Transgressive resistant RILs in our study in both populations, carry resistance allele (at identified loci) from both parents, thus indicating that the intercrossing of moderately resistant or moderately susceptible lines may improve resistance. This approach may be utilized in the future to pyramid multiple number of additive QTL with small effect. The effectiveness of pyramiding genes or QTL regions of small effect is evident in the case of wheat rusts and Fusarium head blight studies (McCartney et al., 2007; von der Ohe et al., 2010; Singh et al., 2011). Molecular markers can be valuable to select loci of interest. The proportion of phenotypic variation explained by identified QTL in our work was small as compared to previously published studies (Singh et al., 2003, 2007; Kaur et al., 2016). This could be attributed to the level of resistance carried by parents in our populations. Both Pop1 and Pop2 utilized parents with moderate resistance/susceptibility, particularly for Pop2. It is also evident from the fact that QTL from susceptible parents were also identified in our work. In Pop2, half of the resistance QTL were contributed by the susceptible parent. It is noteworthy that the two populations have also been evaluated for Septoria tritici blotch, yellow rust, days to heading and plant height (Singh, unpublished data), and none of the SNP identified in the current study for KB resistance showed linkage drag associated with the aforementioned traits.

None of the QTL, except *QKb.cim-3BS1* and *QKb.cim-3BS2*, overlapped in terms of their physical position (**Table 7**). *QKb.cim-3BS1* and *QKb.cim-3BS2* appear to overlap from the physical location of their flanking markers. Of the predicted proteins in our study, protein-kinase (including wall-associated) (Deokar et al., 2018), NAC domain proteins (Deokar et al., 2018), leucine rich repeats (Bent and Mackey, 2007; Deokar et al., 2018), Glutathione S-transferase family proteins and 4-coumarate CoA ligase (Dhokane et al., 2016), ABC transporter (Krattinger et al., 2009) have previously been reported for their role in plant disease resistance and stress responses, thus it is quite likely that they might be one of the candidate proteins for KB resistance. However, the potential candidates listed in **Table 8** must be considered with caution because these not the only proteins predicted in the QTL intervals but a few of many (**Supplementary Table S1**).

Marker-assisted selection is a valuable tool for breeders for the successful introgression of traits with complex phenotypes, such as KB, Fusarium head blight, ascochyta blight in chickpea, etc., into adapted germplasm (Brooks et al., 2006). However, most of the studies in the past have used SSR markers to map loci conferring resistance to KB, and these markers are not always amenable to MAS as they span a large physical distance. Microsatellite or SSR markers detect high level of polymorphism and they often give multiple bands, particularly in polyploids such as wheat (2n = 6x = 42). In cases such as wheat, where homo-, homeo-, or paralogs of genes of interest interfere with marker resolution, SNP markers are desirable. Our study utilized SNP markers to genotype RILs, and the markers can be converted into breeder-friendly KASP markers, which can easily be employed in breeding programs.

## Conclusion

Results from this study suggest that elite lines with moderate resistance to KB possess several genes/QTL with small effect, and have a potential to be used in gene pyramiding for improving resistance. Also, as the lines utilized in our study do not share pedigree with previously reported lines, e.g., HD29, ALDAN “S”/IAS 58 etc., the reported QTL could be novel. Flanking SNP markers can easily be utilized in MAS for introgression of identified QTL regions, even in the absence of phenotyping or at early generations. Additionally, selection of the best resistant lines from both populations can be used for intercrossing to pyramid QTL from all four parents in one background.

## Author Contributions

GB analyzed, interpreted data, and wrote the manuscript. GF-D, CS, XH, RS, and PS collected the data and handled the mapping population. PS conceived and supervised the research. All authors have read and approved the final version of the manuscript.

## Conflict of Interest Statement

The authors declare that the research was conducted in the absence of any commercial or financial relationships that could be construed as a potential conflict of interest.

## Footnotes

1 https://wheat-urgi.versailles.inra.fr/Seq-Repository/Assemblies

## Supplementary Material

TABLE S1 List of annotated genes in Karnal Bunt resistance QTL intervals.
